# Therapeutic targets for lung cancer: genome-wide Mendelian randomization and colocalization analyses

**DOI:** 10.3389/fphar.2024.1441233

**Published:** 2024-10-28

**Authors:** Yi Luan, Desheng Xian, Changwen Zhao, Xin Qing, Hanlin He, Kaixuan Zheng, Wenjun Song, Taijiao Jiang, Wenjian Wang, Chaohui Duan

**Affiliations:** ^1^ Laboratory Testing and Diagnosis Technology Department of Guangzhou National Laboratory, Clinical Laboratory of Sun Yat-sen Memorial Hospital, Guangzhou, Guangdong, China; ^2^ School of Life Science and Technology, ShanghaiTech University, Shanghai, China; ^3^ State Key Laboratory of Chemical Resource Engineering and Beijing Laboratory of Biomedical Materials, Key Laboratory of Biomedical Materials of Natural Macromolecules, Ministry of Education, University of Chemical Technology, Beijing, China; ^4^ Westchina Hospital, Sichuan University, Chengdu, China; ^5^ Guangzhou Medical University, Guangzhou, Guangdong, China; ^6^ School of Life Sciences, Sun Yat-sen University, Guangzhou, Guangdong, China; ^7^ State Key Laboratory of Respiratory Disease, The Key Laboratory of Advanced Interdisciplinary Studies Center, The First Affiliated Hospital of Guangzhou Medical University, Guangzhou, Guangdong, China; ^8^ Department of Thoracic Surgery, Sun Yat-sen Memorial Hospital, Sun Yat-sen University, Guangzhou, Guangdong, China; ^9^ Guangdong Provincial Key Laboratory of Malignant Tumor Epigenetics and Gene Regulation, Sun Yat-sen Memorial Hospital, Sun Yat-sen University, Guangzhou, China; ^10^ Department of Thoracic Surgery, Shenshan Medical Center, Sun Yat-sen Memorial Hospital, Sun Yat-sen University, Shanwei, Guangdong, China

**Keywords:** lung cancer, drug target, Mendelian randomization, GWAS, colocalization analyses

## Abstract

**Background:**

Lung cancer, categorized into non-small cell lung cancer (NSCLC) and small cell lung cancer (SCLC), remains a significant global health challenge. The development of drug resistance and the heterogeneity of the disease necessitate the identification of novel therapeutic targets to improve patient outcomes.

**Methods:**

We conducted a genome-wide Mendelian randomization (MR) and colocalization analysis using a comprehensive dataset of 4,302 druggable genes and cis-expressed quantitative trait loci (cis-eQTLs) from 31,884 blood samples. The study integrated genomic analysis with eQTL data to identify key genes associated with lung cancer risk.

**Results:**

The analysis revealed five actionable therapeutic targets for NSCLC, including LTB4R, LTBP4, MPI, PSMA4, and TCN2. Notably, PSMA4 demonstrated a strong association with both NSCLC and SCLC risks, with odds ratios of 3.168 and 3.183, respectively. Colocalization analysis indicated a shared genetic etiology between these gene expressions and lung cancer risk.

**Conclusion:**

Our findings contribute to precision medicine by identifying druggable targets that may be exploited for subtype-specific lung cancer therapies.

## 1 Introduction

Lung cancer is a major global health problem and is divided into two main histological types: non-small cell lung cancer (NSCLC) and small cell lung cancer (SCLC) (Thai et al., 2021; Li et al., 2023). NSCLC accounts for approximately 85% of all cases and is typically less aggressive than SCLC, which is known for its rapid growth and early metastasis (Leiter et al., 2023). Despite recent advances in targeted therapies and immunotherapies, the emergence of drug resistance remains a major challenge, leading to treatment failure and poor outcomes (Wang et al., 2021; Lv et al., 2021). The emergence of drug resistance, coupled with the heterogeneity of the disease, highlights the urgent need to identify new therapeutic targets to overcome these limitations and improve outcomes in different subtypes of lung cancer (Muthusamy et al., 2022; Rudin et al., 2021). Addressing this unmet need is critical to the development of next-generation therapies that can effectively combat drug resistance and improve patient survival.

The advent of genomics has revolutionized our ability to discover and validate therapeutic targets (Sherman and Salzberg, 2020). In particular, Mendelian randomization (MR) provides a powerful genetic tool for inferring causal relationships between modifiable risk factors and disease outcomes (Sekula et al., 2016; Birney, 2022). By exploiting the natural randomization of genetic variation, MR can mimic the random allocation of interventions in randomized controlled trials, providing strong evidence for potential drug targets (Ference, 2022; Nelson et al., 2015). This approach is particularly applicable to lung cancer research, as genetic susceptibility plays an important role in the etiology of this disease.

In this study, we leveraged the robust framework of MR to delve into the intricate genetic landscape of lung cancer, to uncover novel druggable targets. By seamlessly integrating comprehensive genomic analysis with gene expression quantitative trait loci (eQTL) data (Zhu et al., 2016), we pinpointed key genes whose expression profiles are intricately linked to the risk of lung cancer. It is our aspiration that this work will propel forward the frontiers of personalized medicine in lung cancer, providing a solid foundation for the development of targeted therapeutics capable of transforming patient prognoses.

## 2 Methods

### 2.1 Data sources

We curated a comprehensive dataset of 4,302 druggable genes, annotated under HGNC nomenclature and located on autosomal chromosomes (Schmidt et al., 2020). Prioritizing proximity to the genes of interest, we systematically identified significant cis-eQTLs (false discovery rate (fdr) < 0.05) within a 1 Mb radius of each gene probe. These cis-eQTLs were sourced from resources provided by the eQTLGen Consortium and a meta-analysis of peripheral blood profiles from 31,684 individuals (Chen et al., 2022). To refine our genetic tools, we selected cis-eQTLs within 100 kb of the genomic position of each gene, resulting in a dataset of eQTLs for 2,405 targetable genes. Leveraging the comprehensive R10 dataset from the Finngen consortium (Kurki et al., 2023), our studies have illuminated the genetic landscape of NSCLC and SCLC. With a rigorous examination of over 300,000 individuals, we have identified a discrete set of 5,315 NSCLC cases juxtaposed with a control group of 314,193, and for SCLC, 717 cases were meticulously compared against the same control cohort.

### 2.2 Drug target identification

**FIGURE 1:**
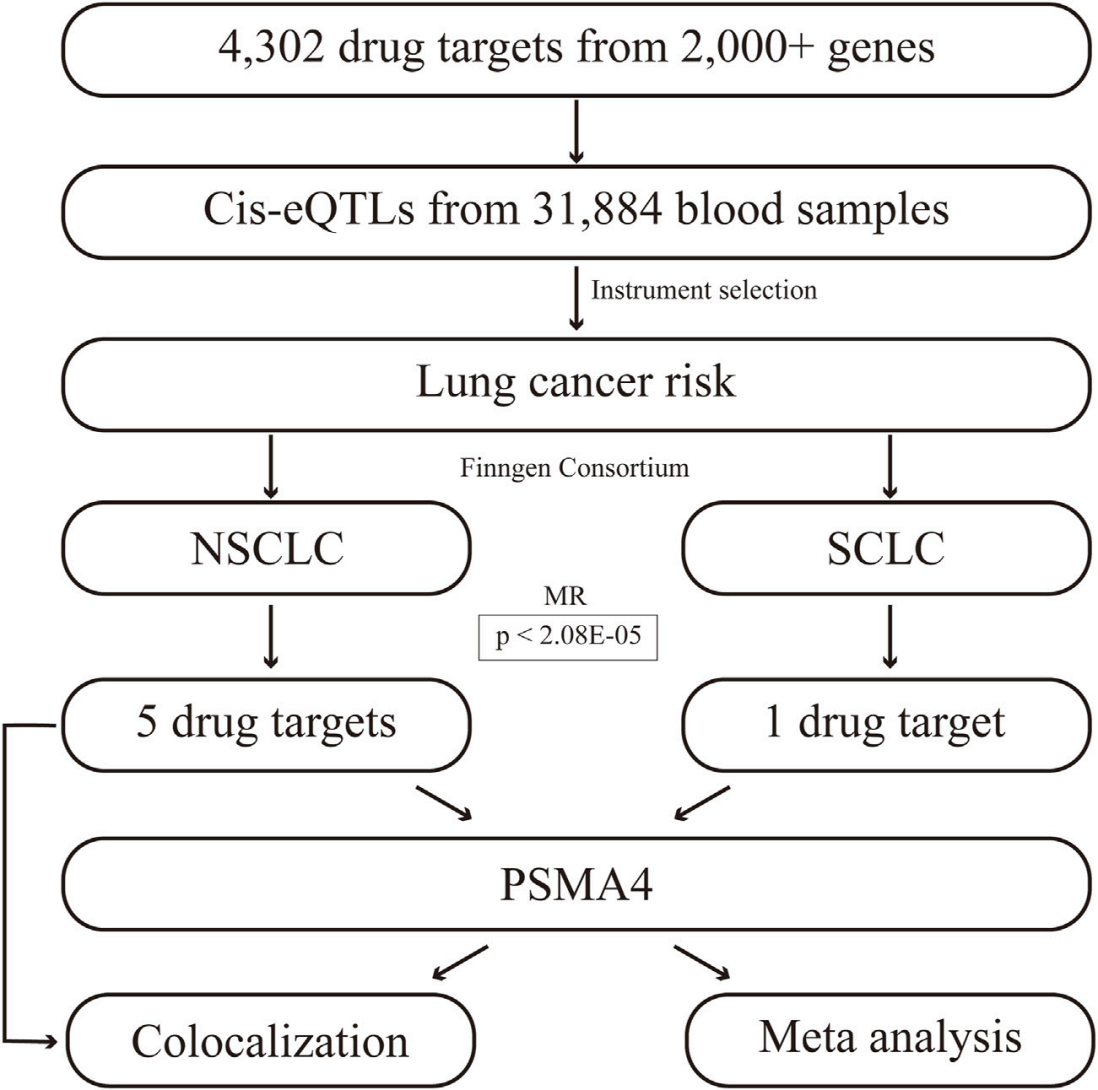
The workflow of this study.

The workflow of this study is illustrated in Figure 1. Initially, we focused on NSCLC and SCLC, recognizing the distinct biological behaviors and clinical implications of these subtypes. The MR analysis was conducted using a TwosampleMR package (Qing et al., 2024), ensuring methodological rigor and comparability of our results. This study adheres to three core assumptions: the genetic variants are relevant and robustly associated with gene expression levels implicated in lung cancer risk; they satisfy the exclusion restriction, indicating no confounding pathways to the disease outcome; and they exhibit sufficient instrument strength to detect a causal effect. We applied stringent filtering criteria to select high-quality genetic instruments, eliminating single nucleotide polymorphisms (SNPs) with an F-statistic below 10 and ensuring conditional independence with no linkage disequilibrium (r^2^ < 0.1). MR estimates were determined using the Wald ratio method for each SNP, followed by meta-analysis using the inverse variance weighted (IVW), MR-Egger, and weighted median models to account for multiple instruments. The IVW approach assumes that all genetic tools are valid and unbiased, and detects causal effects by weighting each SNP by the inverse of its variance to maximize statistical power. The IVW method provides conventional causal estimates under the assumption of no pleiotropy, whereas the MR-Egger method provides a robustness test for the effects of potential pleiotropy, ensuring that our causal inferences are not subject to pleiotropy bias (Chen et al., 2023; Chen et al., 2024). The MR-Egger regression was specifically employed to address potential pleiotropy when instruments had more than two variants. Heterogeneity among instruments was assessed using Cochran’s Q test. To correct for multiple testing, we applied Bonferroni adjustments and fdr to set significance thresholds. Additionally, we further applied transcriptome analysis to verify the reliability of the current results based on TCGA and GEO databases, and the detailed methods were presented in our previous publications (Qing et al., 2022; Qing et al., 2023). TCGA_LUAD and GSE19188, as lung cancer datasets, were included in this study.

### 2.3 Colocalization and meta-analysis

To identify therapeutic targets for lung cancer, we conducted a meta-analysis to aggregate the effects of genes significant for both NSCLC and SCLC, elucidating their concrete manifestations in lung cancer pathology (Cheung and Vijayakumar, 2016). The colocalization analysis was performed using the coloc R package with default priors (Giambartolomei et al., 2018), focusing on the relationship between MR-identified targets and lung cancer risk. We established the prior likelihood for cis-eQTL (H1) and lung cancer connections (H2) at 1E−04, and the probability of a single variant influencing both traits (H4) at 1E−05. A posterior probability (PPH4) exceeding 0.70 indicated significant colocalization, thereby identifying genes with strong colocalization signals as potential therapeutic targets for lung cancer. We further used meta package to identify the comprehensive role of PSMA4 in lung cancer (Balduzzi et al., 2019).

## 3 Results

### 3.1 Actionable therapeutic targets for NSCLC

**FIGURE 2:**
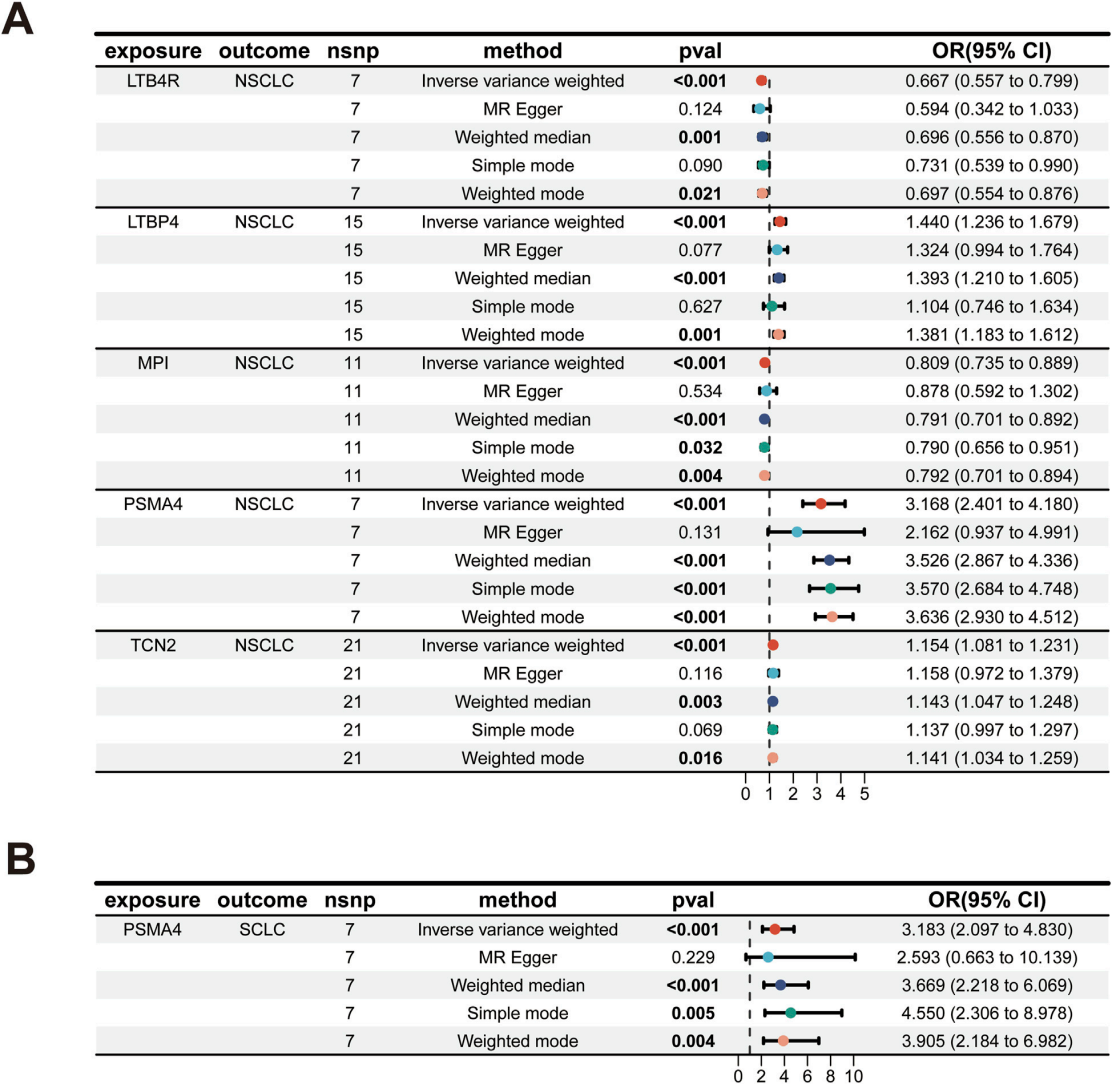
Significant druggable biomarkers for lung cancer. **(A)** NSCLC. **(B)** SCLC.

Our analysis has successfully identified five actionable therapeutic targets associated with NSCLC (Figure 2A; Supplementary Table S1). The analysis was conducted using various statistical methods, including IVW, MR Egger, weighted median, simple mode, and weighted mode, to ensure robustness and reduce the potential for bias (Birney, 2022; Yuan et al., 2023a; Yarmolinsky et al., 2022). The genetic biomarkers LTB4R, LTBP4, MPI, PSMA4, and TCN2 demonstrated significant associations with NSCLC risk. Notably, PSMA4 emerged as a prominent target, with an odds ratio (OR) of 3.168 (95% CI 2.401–4.180), indicating a strong association with NSCLC risk. This finding was highly significant, with a *p*-value less than 0.001, even after Bonferroni and false discovery rate (FDR) adjustments, underscoring its potential as a therapeutic target (Yuan et al., 2021). The IVW method provided consistent results for LTB4R (OR 0.667, 95% CI 0.557–0.799), LTBP4 (OR 1.440, 95% CI 1.236–1.679), MPI (OR 0.809, 95% CI 0.735–0.889), and TCN2 (OR 1.154, 95% CI 1.081–1.231), all of which showed significant associations with NSCLC risk post-adjustment for multiple testing (Table 1). To assess the robustness of our MR findings, we conducted pleiotropy testing using Cochran’s Q test and MR-Egger intercept. In our analysis, we observed significant heterogeneity, as indicated by Cochran’s Q test (Q = 23.776, *p*-value = 0.001), which suggests that the effect of genetic variants on PSMA4 expression might vary across the study population. However, the absence of pleiotropy is supported by the non-significant MR-Egger intercept (*p*-value = 0.386), indicating that the observed associations are not likely due to pleiotropic effects where the genetic variants influence both the exposure (PSMA4 expression) and the outcome (NSCLC risk) in a biased manner (Supplementary Table S2). For LTB4R, MPI, and TCN2, no significant heterogeneity was observed (*p* > 0.05), supporting their potential causal roles in NSCLC.

**TABLE 1:** Genetically predicted biomarkers on the risk of lung cancer.

Exposure	Outcome	Method	OR (95% CI)	p-adjust (Bonferroni)	p-adjust (FDR)	Cochran’ Q test		MR-Egger	
						Q	*p*-value	Intercept	*p*-value
LTB4R	NSCLC	IVW	0.667 (0.557–0.799)	0.027	0.007	4.356	0.629	0.013	0.682
LTBP4	NSCLC	IVW	1.44 (1.239–1.679)	0.007	0.004	27.655	0.016	0.014	0.506
MPI	NSCLC	IVW	0.809 (0.735–0.889)	0.029	0.007	4.569	0.918	−0.021	0.683
PSMA4	NSCLC	IVW	3.168 (2.401–4.180)	<0.001	<0.001	23.776	0.001	0.064	0.386
TCN2	NSCLC	IVW	1.154 (1.081–1.231)	0.041	0.008	11.369	0.936	−0.001	0.965
PSMA4	SCLC	IVW	3.183 (2.097–4.83)	<0.001	<0.001	7.713	0.26	0.034	0.768

Note: IVW: Inverse-variance weighted; NSCLC: Non-small cell lung cancer; OR: odds ratio; SCLC: small cell lung cancer.

### 3.2 Actionable therapeutic target for SCLC

MR analysis has pinpointed PSMA4 as a significant actionable therapeutic target (Figure 2B; Supplementary Table S1). The OR for the association between PSMA4 and SCLC risk was 3.183 (95% CI 2.097–4.830), with a *p*-value less than 0.001, indicating a strong and significant relationship. This target was also significant after adjustments for multiple tests (Table 1), reinforcing its potential as a therapeutic intervention for SCLC. The robustness of this association was further supported by the lack of significant heterogeneity in Cochran’s Q test (*p*-value = 0.26) and no evidence of directional pleiotropy in the MR-Egger intercept (*p*-value = 0.768, Supplementary Table S2). Meanwhile, we observed that LTB4R, LTBP4, MPI, and TCN2 did not demonstrate a significant association with SCLC, with adjusted *p*-values greater than 0.05.

### 3.3 Colocalization analysis

**FIGURE 3:**
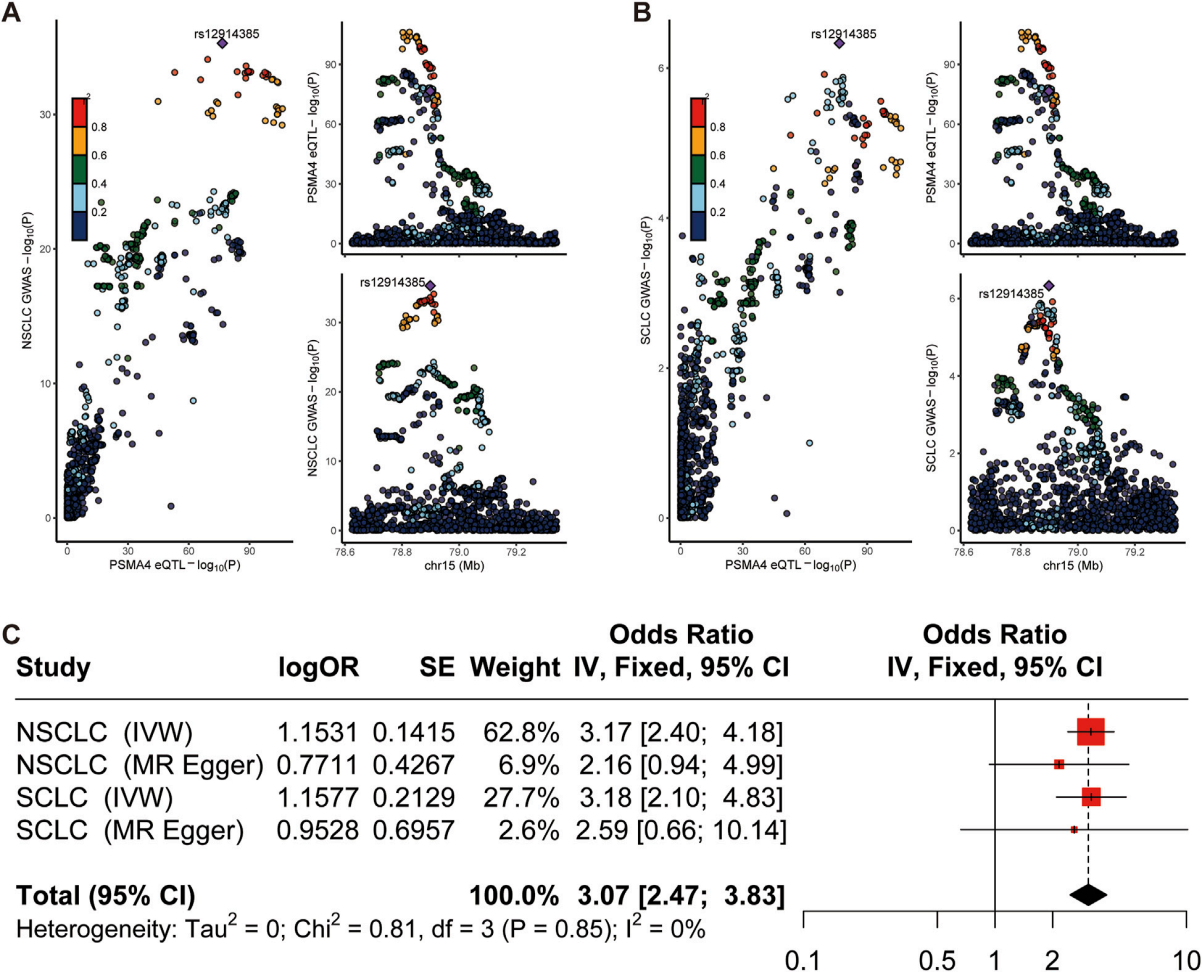
Colocalization and meta-analysis. **(A, B)** Regional plot of colocalization evidence of PSMA4 and NSCLC/SCLC susceptibility. **(C)** Meta analysis result.

To elucidate the potential shared causal genetic variants between lung cancer risk and gene expression levels, we conducted a meticulous colocalization analysis (Yuan et al., 2023b). This analysis was designed to assess whether the same underlying genetic factors might drive SNPs associated with lung cancer and eQTLs. Our findings pointed towards a probable common causal variant in the region of LTBP4, implicating a shared genetic etiology between LTBP4 expression and NSCLC risk (posterior probability of causality, PP.H4 = 0.77; Supplementary Figure S1; Supplementary Table S3). This was corroborated by the colocalization signal observed for PSMA4 and SCLC, with a strong posterior probability (PP.H4 = 0.84; Figures 3A, B), suggesting that genetic variation in the PSMA4 region may be linked to both gene expression and SCLC risk. The colocalization plots visually represent the correspondence between eQTL and GWAS signals, with the r^2^ values indicating the proportion of variance explained by the shared causal variant. The systematic approach of combining MR with colocalization analyses has bolstered our discovery of these genes as potential drug targets, underscored by robust evidence of a shared genetic influence on gene expression and lung cancer risk (Zeng et al., 2024). To delineate the role of PSMA4 in lung cancer more distinctly, the integrated results from this meta-analysis further substantiate the notion that PSMA4 is a promising therapeutic target in lung cancer, offering a potential avenue for targeted treatment strategies (Figure 3C). The colocalization analysis showed high confidence in shared genetic variants driving both gene expression and cancer risk. These findings bolster the relevance of genes like PSMA4 as potential drug targets in both NSCLC and SCLC.

### 3.4 Replication analyses

In this study, we found that the expression level of PSMA4 was significantly upregulated in lung cancer by comparative analysis of lung cancer tissues and normal tissues (Supplementary Figure S2). Specifically, in the TCGA_LUAD dataset, the overall survival of patients was significantly higher in the PSMA4 high-expression group compared to the low-expression group (Wilcoxon test, *p* = 4.3e−08), while a similar trend was observed in the GSE19188 dataset (Wilcoxon test, *p* = 2.5e−06). Furthermore, analyzing the survival curves by Log-rank test, we found that in the TCGA_LUAD dataset, there was a significant difference in survival time between patients with high and low PSMA4 expression (Log-rank test, *p* = 0.0029), whereas in the GSE19188 dataset, although the correlation between the PSMA4 expression level and survival did not reach statistical significance (Log-rank test, *p* = 0.55), but the overall trend was consistent with the TCGA_LUAD dataset. These results suggest that PSMA4 may play an important role in the development of lung cancer, and changes in its expression level may serve as a potential biomarker for predicting the prognosis of lung cancer patients.

## 4 Discussion

Our study presents a comprehensive analysis leveraging MR and colocalization approaches to identify novel therapeutic targets for lung cancer. The identification of PSMA4 as a significant target for both NSCLC and SCLC is particularly noteworthy. The robustness of our findings is evidenced by the significant odds ratios observed, which persisted after stringent adjustments for multiple tests.

The presence of heterogeneity in the MR analysis for PSMA4, without evidence of directional pleiotropy, suggests that while there may be variation in the effect of genetic instruments, the overall association with lung cancer risk is not biased (Birney, 2022; Yuan et al., 2022; Papadimitriou et al., 2020). This is further supported by the consistent results across different MR methods, reinforcing the reliability of PSMA4 as a therapeutic target. The colocalization analysis provides compelling evidence that the genetic variants associated with PSMA4 expression are likely to be in causal pathways related to lung cancer risk. The posterior probabilities of causality exceeding the threshold for SCLC substantiate the potential of PSMA4 in targeted therapies.

Our findings are in concordance with the existing literature that implicates PSMA4 in cancer biology (Chiao et al., 2021; Amos et al., 2010; O’Brien et al., 2018). Previous studies have observed an association between the PSMA4 gene region and lung cancer risk, which is in line with our MR and colocalization analysis results (Amos et al., 2010; Wang et al., 2015; Pintarelli et al., 2017; Bai et al., 2022). PSMA4 is a core component of the proteasome 19S regulatory granule, which plays a key role in protein hydrolysis by regulating the unfolding and translocation of ubiquitinated proteins into the proteasome core for degradation. This protein hydrolysis function is essential for the maintenance of protein homeostasis, particularly in cancer cells, where elevated proteasome activity supports rapid cell proliferation by degrading misfolded or damaged proteins that accumulate during increased metabolic activity (Chiao et al., 2021; Liu et al., 2009). Liu Y, et al. reported that PSMA4 mRNA levels are higher in lung tumor tissues compared to normal lung tissues, indicating a role for PSMA4 in promoting cancer cell proliferation (Liu et al., 2009). This is corroborated by O’Brien TD, et al., who discussed PSMA4 as one of the few genes shared between SCLC and NSCLC, highlighting its potential role in the pathogenesis of lung cancer (O’Brien et al., 2018).

The enhanced understanding of PSMA4’s functional role in lung cancer provided by our study, combined with these previous findings, offers a strong rationale for exploring its targeting in therapeutic interventions. PSMA4 is involved in the regulation of the cell cycle, particularly through the degradation of cell cycle proteins and cell cycle protein-dependent kinase inhibitors. By regulating key cell cycle proteins, PSMA4 ensures that cancer cells bypass normal checkpoints, thereby promoting uncontrolled proliferation (Wang et al., 2015). In addition, by participating in the degradation of immunomodulatory proteins, PSMA4 may influence the tumor immune microenvironment by regulating the expression of tumor antigens and evading immune surveillance mechanisms (Kakumu et al., 2017). Given PSMA4’s involvement in proteolysis—a process critical for protein turnover, cell cycle regulation, and immune response modulation—its modulation could present a novel therapeutic strategy (Xia et al., 2024). This could involve the development of small molecule inhibitors, monoclonal antibodies, or other modalities designed to interfere with the proteolytic function of PSMA4, potentially slowing tumor progression or enhancing the efficacy of existing treatments.

Our findings contribute to the burgeoning field of precision medicine in oncology by offering potential targets that could be exploited for the development of subtype-specific therapies. The identification of LTB4R, LTBP4, MPI, and TCN2, in conjunction with PSMA4, highlights the intricate heterogeneity of lung cancer and underscores the necessity for tailored therapeutic strategies. LTB4R’s role in mediating inflammatory responses in lung cancer, as demonstrated by Jala VR et al., suggests its potential as an anti-inflammatory therapeutic target (Jala et al., 2017). The association of LTBP4 genetic variants with patient outcomes, as shown by Shin KM et al., indicates the value of genetic profiling in prognostic assessments (Shin et al., 2018). Although MPI’s direct link to lung cancer requires further elucidation, its metabolic functions hint at a role in cancer metabolism (Yao et al., 2021). The potential involvement of TCN2 in iron metabolism dysregulation in cancer offers a novel perspective on cancer cell biology (Oussalah et al., 2017). These findings collectively pave the way for the development of subtype-specific treatments, heralding a new era in personalized medicine for lung cancer patients.

It is important to acknowledge that while our study provides strong genetic evidence for the role of these targets in lung cancer, functional validation, and clinical trials are necessary to realize their therapeutic potential. Our study has some limitations. Firstly, although our study includes a large cohort, the generalizability of our findings to other populations with different genetic backgrounds may be limited. Secondly, our study’s retrospective design limits our ability to establish temporal relationships and causality. We recommend that future research employ prospective study designs to further investigate the causal mechanisms by which these targets contribute to lung cancer development. Thirdly, the genetic markers we have identified are correlated with lung cancer risk; however, we acknowledge that unmeasured confounders could influence this relationship. Future research should focus on elucidating the biological mechanisms by which these targets influence lung cancer risk and progression.

## 5 Conclusion

In conclusion, our genome-wide MR and colocalization analyses have successfully identified PSMA4 as a promising therapeutic target for lung cancer. The robust associations observed, coupled with the supportive colocalization signals, highlight the potential of PSMA4 in the development of targeted lung cancer therapies. Additionally, the identification of several actionable targets for NSCLC emphasizes the heterogeneity of the disease and the value of personalized medicine approaches.

## Data Availability

The original contributions presented in the study are included in the article/Supplementary Material, further inquiries can be directed to the corresponding authors.
