# Supplementary material for: Therapeutic targets for lung cancer: genome-wide Mendelian randomization and colocalization analyses
Source: Front Pharmacol. 2024 Oct 28;15:1441233. doi: 10.3389/fphar.2024.1441233 (PMC11551539; doi:10.3389/fphar.2024.1441233)
Supplement: Supplementary file 1 [file DataSheet1.PDF]

Figure S1: Colocalization plots of significant biomarkers.

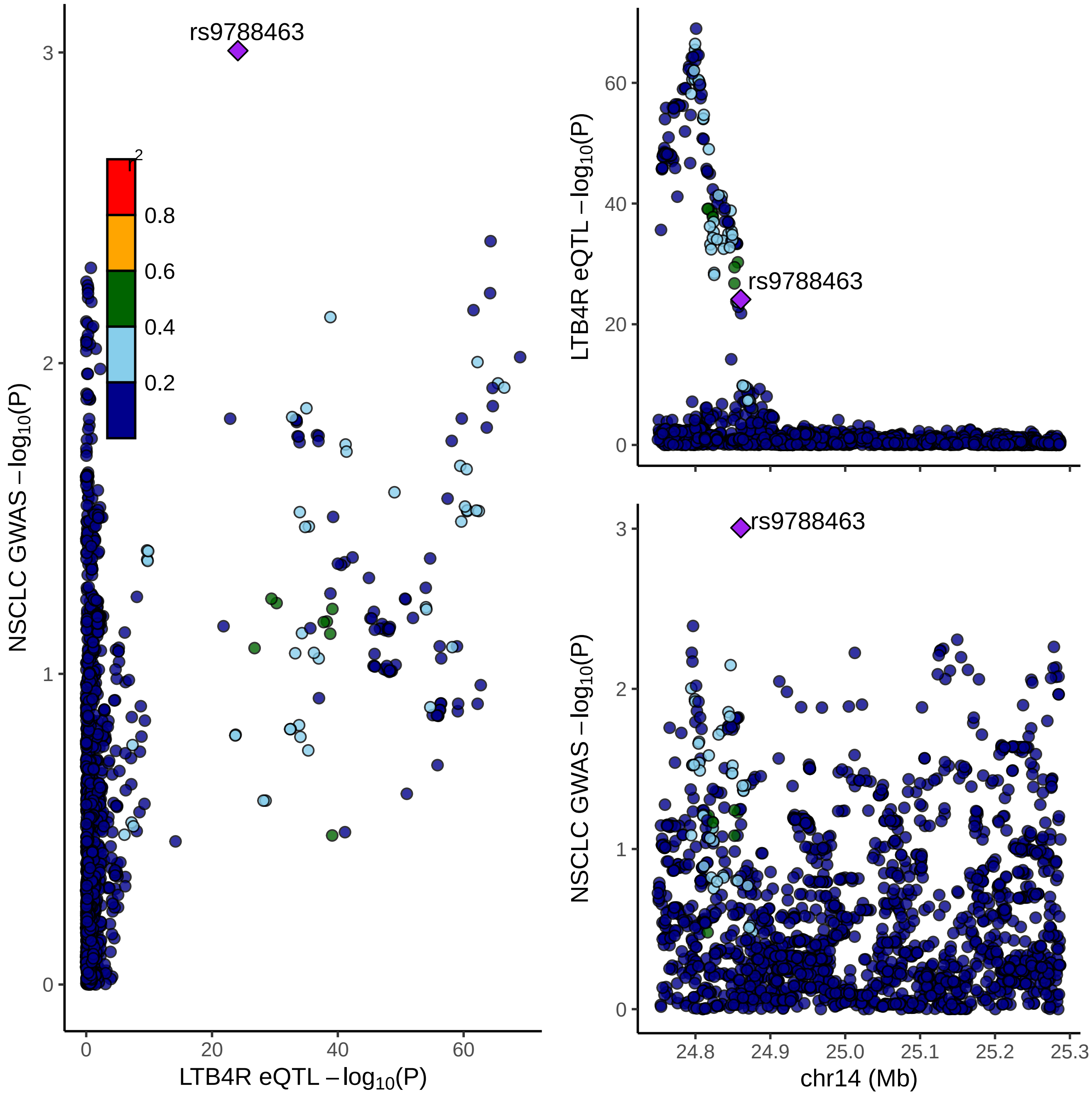

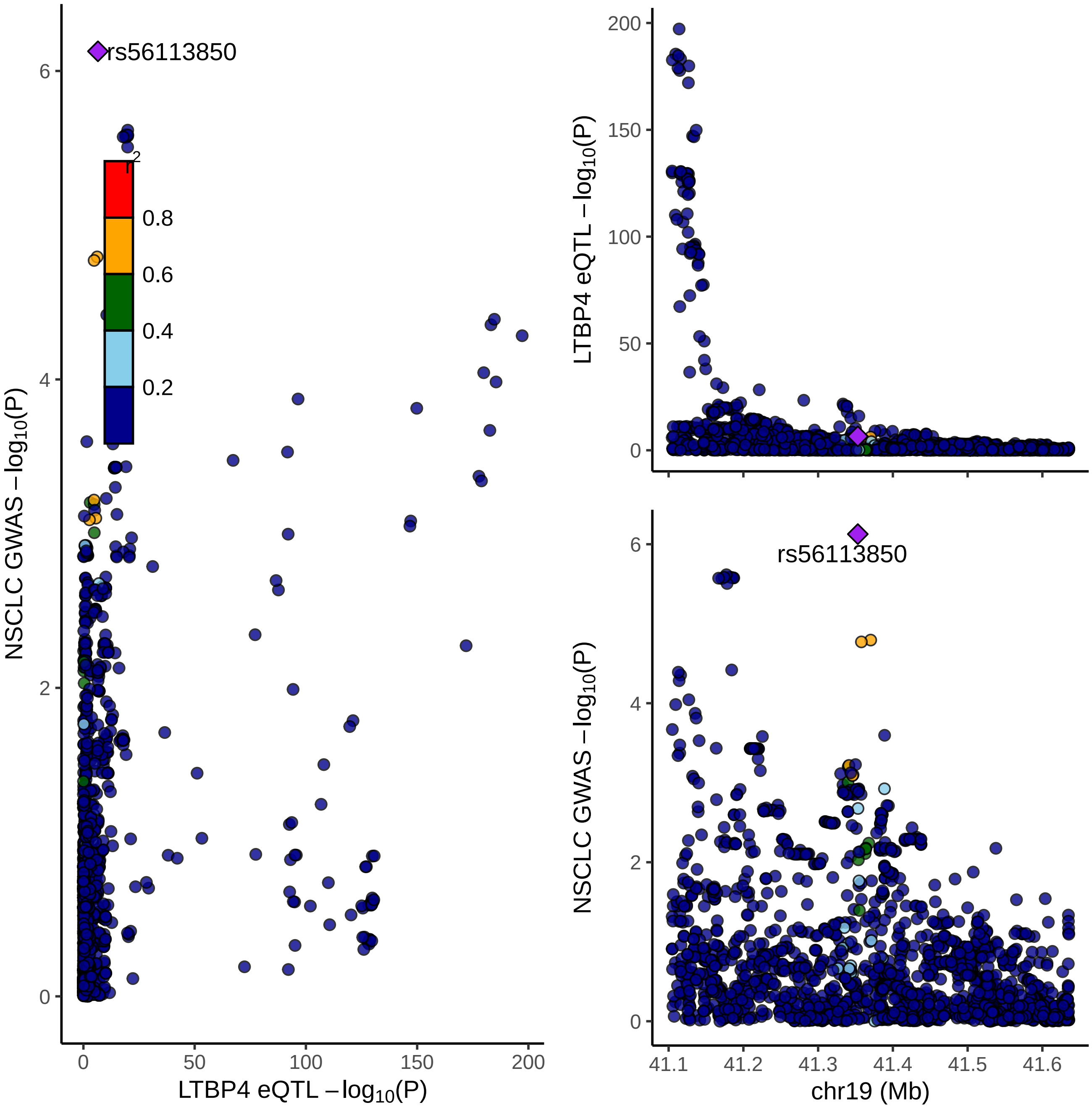

rs56113850
r2
0.8
0.6
0.4
0.2
NSCLC GWAS −log10(P)
LTBP4 eQTL −log10(P)
0
50
100
150
200
2
4
6
41.1
41.2
41.3
41.4
41.5
41.6
chr19 (Mb)
rs56113850

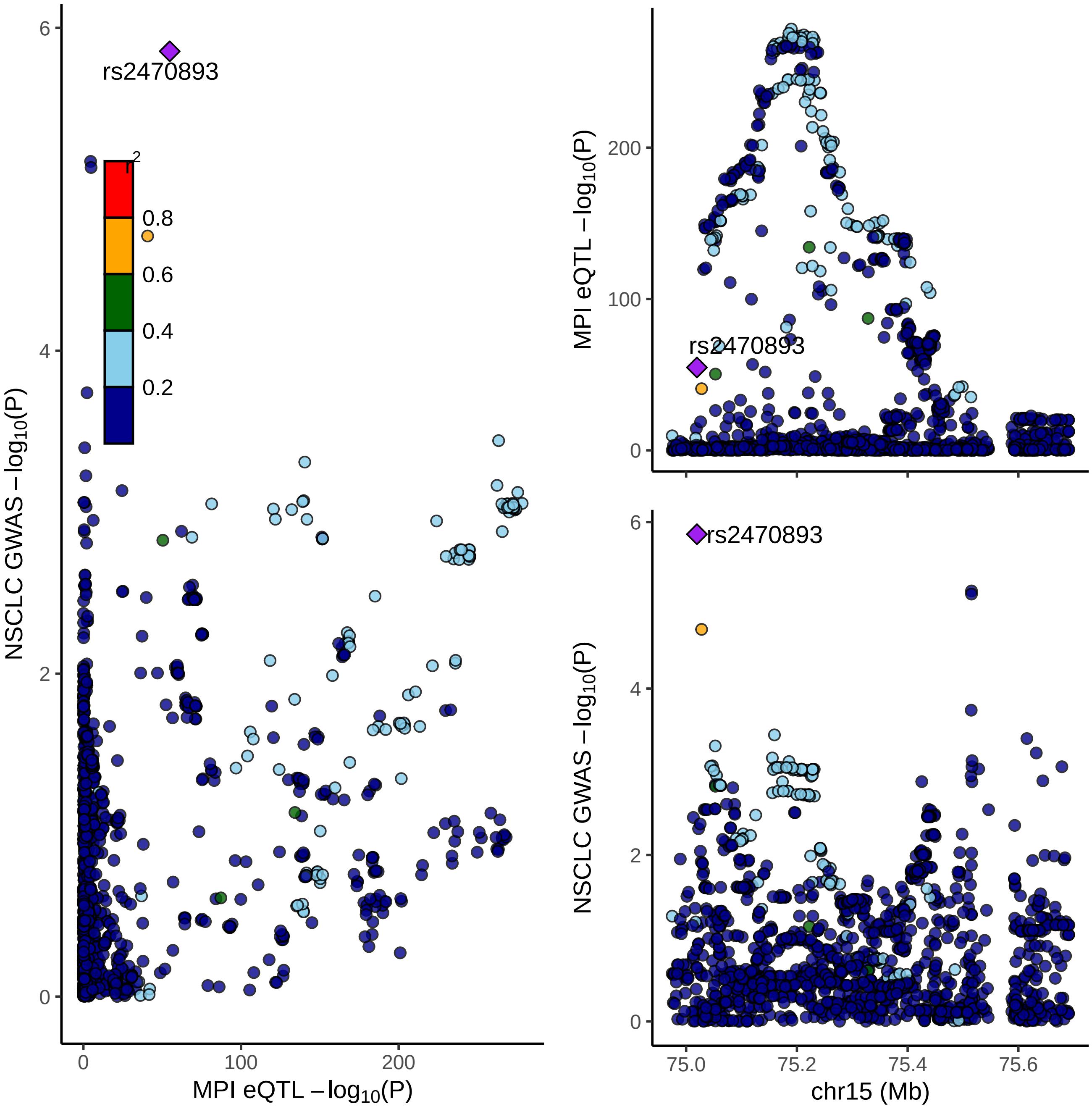

rs2470893
r2
0.8
0.6
0.4
0.2
NSCLC GWAS – log10(P)
MPI eQTL – log10(P)
chr15 (Mb)

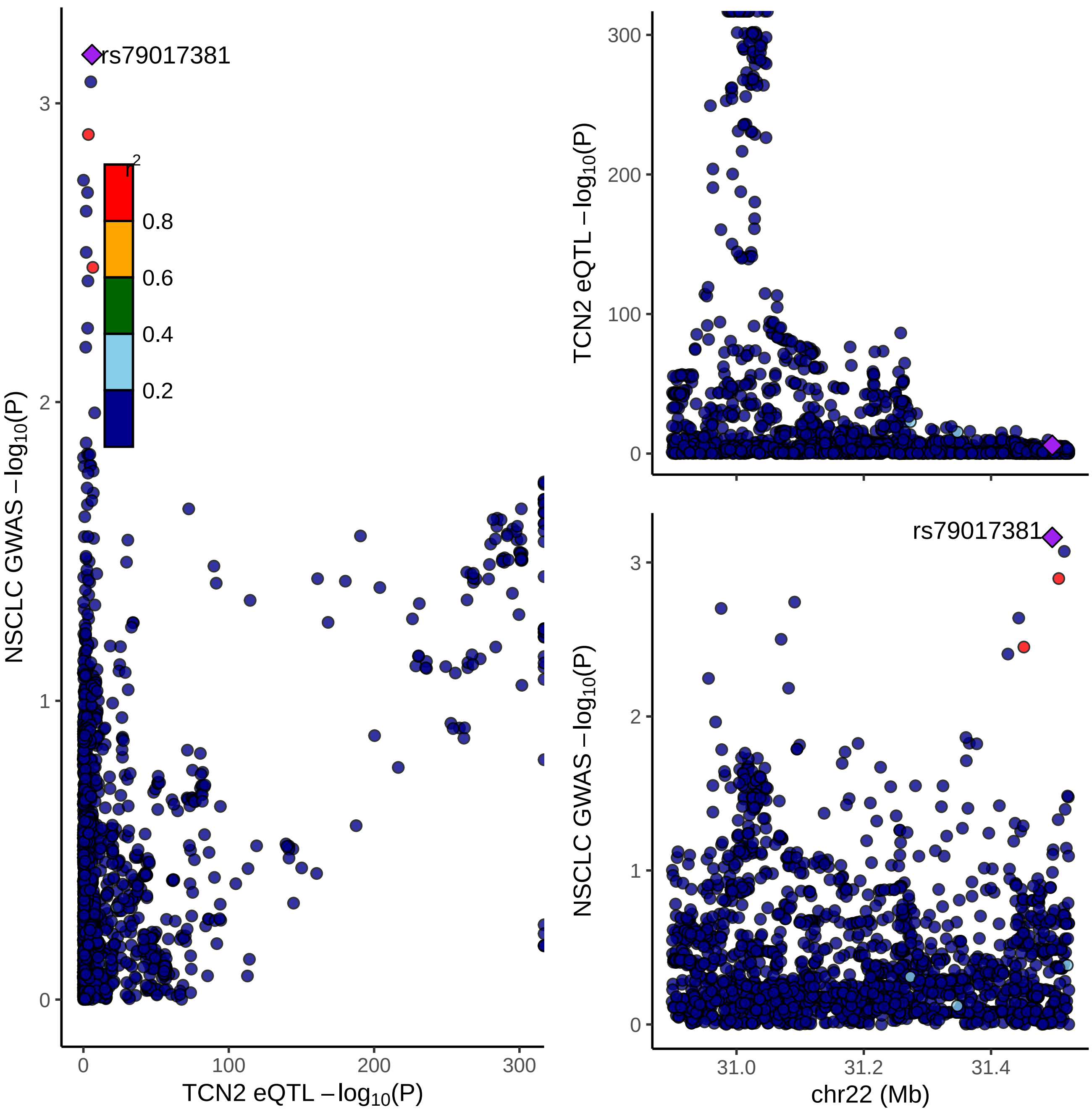

rs79017381
NSCLC GWAS −log10(P)
TCN2 eQTL −log10(P)
r2
0.8
0.6
0.4
0.2
0
1
2
3
100
200
300
TCN2 eQTL −log10(P)
NSCLC GWAS −log10(P)
rs79017381
31.0
31.2
31.4
chr22 (Mb)

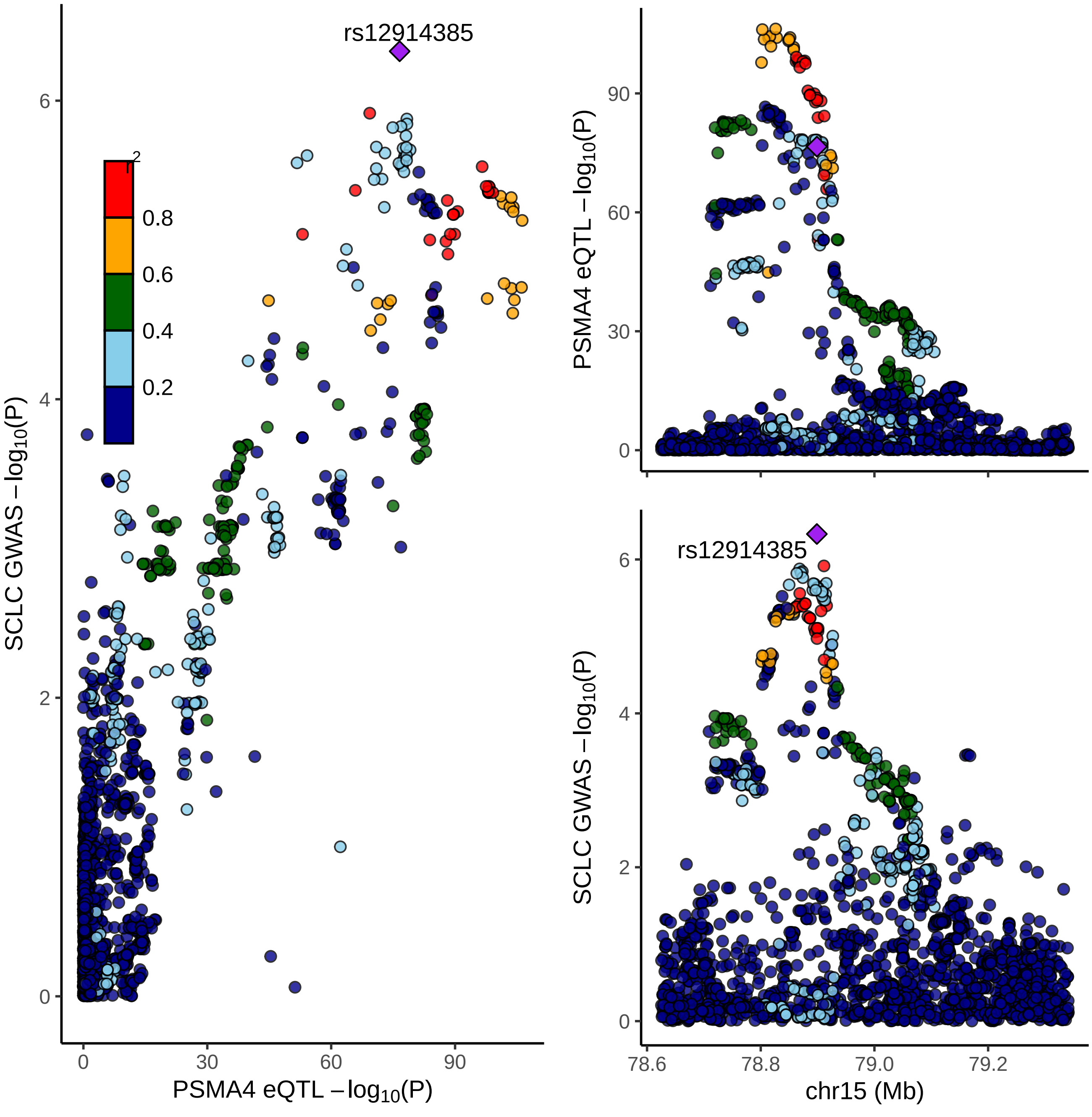
rs12914385
SCLC GWAS –log10(P)
PSMA4 eQTL –log10(P)
r2
0.8
0.6
0.4
0.2
chr15 (Mb)
78.6
78.8
79.0
79.2

Figure S2: Expression analyses of PSMA4 in lung cancer tissue based on TCGA and GEO.

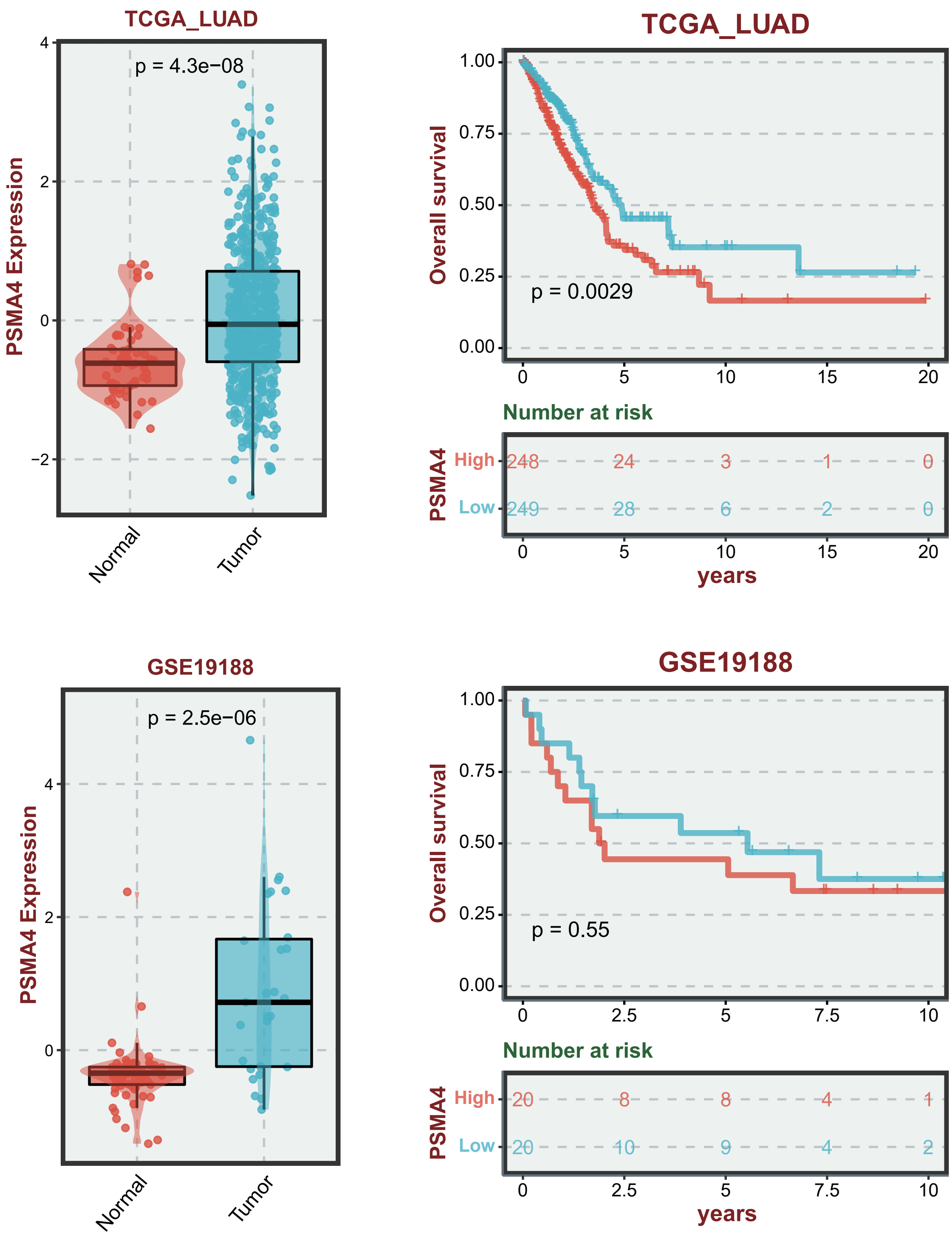

Table S1. The MR results of druggable gene in lung cancer.

outcome	exposure	method	nsnp	b	se	pval	lo_ci	up_ci	or	or_lci95	or_uci95	estimate	p_adj_bon	p_adj_fdr
SCLC	PSMA4	MR Egger	7	0.9528	0.695731	2.29e-01	-0.41083	2.316433	2.592961	0.663098	10.13944	2.593 (0.6631-10.1394)		
SCLC	PSMA4	Weighted n	7	1.299871	0.256825	4.16e-07	0.796495	1.803248	3.668825	2.217754	6.069329	3.6688 (2.2178-6.0693)		
SCLC	PSMA4	Inverse var	7	1.157675	0.212857	5.37e-08	0.740475	1.574876	3.182526	2.096931	4.830141	3.1825 (2.0969-4.8301)	0.000132004	0.000132
SCLC	PSMA4	Simple moc	7	1.515221	0.346688	4.72e-03	0.835712	2.194729	4.550425	2.306457	8.977568	4.5504 (2.3065-8.9776)		
SCLC	PSMA4	Weighted n	7	1.362331	0.296464	3.71e-03	0.781262	1.943401	3.905287	2.184226	6.982457	3.9053 (2.1842-6.9825)		
SCLC	LTBP4	MR Egger	15	0.086028	0.269527	7.55e-01	-0.44225	0.614301	1.089837	0.642592	1.848365	1.0898 (0.6426-1.8484)		
SCLC	LTBP4	Weighted n	15	0.058773	0.189884	7.57e-01	-0.3134	0.430945	1.060535	0.730958	1.538711	1.0605 (0.731-1.5387)		
SCLC	LTBP4	Inverse var	15	0.131352	0.146446	3.70e-01	-0.15568	0.418386	1.140369	0.855832	1.519507	1.1404 (0.8558-1.5195)	1	0.84018
SCLC	LTBP4	Simple moc	15	0.161049	0.321036	6.24e-01	-0.46818	0.79028	1.174742	0.62614	2.204013	1.1747 (0.6261-2.204)		
SCLC	LTBP4	Weighted n	15	0.062559	0.191468	7.49e-01	-0.31272	0.437836	1.064557	0.731456	1.549351	1.0646 (0.7315-1.5494)		
SCLC	MPI	MR Egger	11	0.521722	0.76451	5.12e-01	-0.97672	2.020163	1.684927	0.376545	7.539552	1.6849 (0.3765-7.5396)		
SCLC	MPI	Weighted n	11	0.141864	0.168161	3.99e-01	-0.18773	0.47146	1.15242	0.828837	1.602333	1.1524 (0.8288-1.6023)		
SCLC	MPI	Inverse var	11	0.171731	0.177002	3.32e-01	-0.17519	0.518655	1.187359	0.839295	1.679767	1.1874 (0.8393-1.6798)	1	0.814506
SCLC	MPI	Simple moc	11	0.320854	0.253806	2.35e-01	-0.17661	0.818314	1.378304	0.83811	2.266674	1.3783 (0.8381-2.2667)		
SCLC	MPI	Weighted n	11	0.148623	0.179468	4.27e-01	-0.20313	0.50038	1.160235	0.816168	1.649348	1.1602 (0.8162-1.6493)		
SCLC	TCN2	MR Egger	21	0.279973	0.267799	3.09e-01	-0.24491	0.804859	1.323094	0.782772	2.236382	1.3231 (0.7828-2.2364)		
SCLC	TCN2	Weighted n	21	-0.02534	0.124582	8.39e-01	-0.26952	0.21884	0.974977	0.763745	1.244632	0.975 (0.7637-1.2446)		
SCLC	TCN2	Inverse var	21	0.033158	0.099351	7.39e-01	-0.16157	0.227886	1.033713	0.850806	1.255943	1.0337 (0.8508-1.2559)	1	0.956673
SCLC	TCN2	Simple moc	21	0.008174	0.201633	9.68e-01	-0.38703	0.403375	1.008208	0.679073	1.496868	1.0082 (0.6791-1.4969)		
SCLC	TCN2	Weighted n	21	-0.0434	0.142943	7.65e-01	-0.32357	0.236767	0.957527	0.723561	1.267146	0.9575 (0.7236-1.2671)		
SCLC	LTB4R	MR Egger	7	0.280447	0.751628	7.24e-01	-1.19274	1.753638	1.323721	0.303388	5.775578	1.3237 (0.3034-5.7756)		
SCLC	LTB4R	Weighted n	7	-0.31064	0.303033	3.05e-01	-0.90459	0.283301	0.732975	0.404709	1.327504	0.733 (0.4047-1.3275)		
SCLC	LTB4R	Inverse var	7	-0.44533	0.245768	7.00e-02	-0.92704	0.036371	0.640611	0.395724	1.037041	0.6406 (0.3957-1.037)	1	0.524732
SCLC	LTB4R	Simple moc	7	-0.19404	0.420953	6.61e-01	-1.01911	0.631026	0.823624	0.360916	1.879539	0.8236 (0.3609-1.8795)		
SCLC	LTB4R	Weighted n	7	-0.26072	0.323222	4.51e-01	-0.89424	0.372795	0.770496	0.40892	1.451786	0.7705 (0.4089-1.4518)		
NSCLC	PSMA4	MR Egger	7	0.771136	0.426739	1.31e-01	-0.06527	1.607544	2.162221	0.936813	4.990538	2.1622 (0.9368-4.9905)		
NSCLC	PSMA4	Weighted n	7	1.26019	0.105539	7.27e-33	1.053334	1.467046	3.526092	2.867196	4.336405	3.5261 (2.8672-4.3364)		
NSCLC	PSMA4	Inverse var	7	1.15308	0.141455	3.59e-16	0.875829	1.430331	3.167934	2.400864	4.180081	3.1679 (2.4009-4.1801)	8.83549E-13	8.84E-13
NSCLC	PSMA4	Simple moc	7	1.272643	0.145486	1.24e-04	0.98749	1.557796	3.570275	2.684488	4.748342	3.5703 (2.6845-4.7483)		
NSCLC	PSMA4	Weighted n	7	1.290893	0.1101	2.32e-05	1.075097	1.506689	3.636032	2.930277	4.511769	3.636 (2.9303-4.5118)		
NSCLC	LTBP4	MR Egger	15	0.280984	0.14632	7.70e-02	-0.0058	0.567771	1.324432	0.994214	1.764329	1.3244 (0.9942-1.7643)		
NSCLC	LTBP4	Weighted n	15	0.331695	0.072162	4.30e-06	0.190258	0.473132	1.393327	1.209561	1.605013	1.3933 (1.2096-1.605)		
NSCLC	LTBP4	Inverse var	15	0.36498	0.078125	2.99e-06	0.211856	0.518105	1.440485	1.235969	1.678843	1.4405 (1.236-1.6788)	0.007346349	0.003673
NSCLC	LTBP4	Simple moc	15	0.09917	0.199865	6.27e-01	-0.29256	0.490905	1.104254	0.746347	1.633794	1.1043 (0.7463-1.6338)		
NSCLC	LTBP4	Weighted n	15	0.323046	0.078922	1.10e-03	0.168358	0.477734	1.381329	1.183361	1.612417	1.3813 (1.1834-1.6124)		
NSCLC	MPI	MR Egger	11	-0.13013	0.201134	5.34e-01	-0.52435	0.264095	0.877982	0.591939	1.302251	0.878 (0.5919-1.3023)		
NSCLC	MPI	Weighted n	11	-0.23493	0.061346	1.28e-04	-0.35517	-0.1147	0.790624	0.701053	0.891638	0.7906 (0.7011-0.8916)		
NSCLC	MPI	Inverse var	11	-0.21248	0.048463	1.16e-05	-0.30747	-0.11749	0.808576	0.735307	0.889147	0.8086 (0.7353-0.8891)	0.028614843	0.007154
NSCLC	MPI	Simple moc	11	-0.23598	0.094799	3.20e-02	-0.42179	-0.05018	0.789796	0.655874	0.951062	0.7898 (0.6559-0.9511)		
NSCLC	MPI	Weighted n	11	-0.23379	0.062161	3.72e-03	-0.35563	-0.11195	0.791528	0.700735	0.894086	0.7915 (0.7007-0.8941)		
NSCLC	TCN2	MR Egger	21	0.146658	0.089175	1.16e-01	-0.02812	0.32144	1.157958	0.972267	1.379112	1.158 (0.9723-1.3791)		
NSCLC	TCN2	Weighted n	21	0.133944	0.044771	2.77e-03	0.046194	0.221695	1.143329	1.047277	1.248191	1.1433 (1.0473-1.2482)		
NSCLC	TCN2	Inverse var	21	0.142991	0.033227	1.68e-05	0.077865	0.208116	1.153719	1.080977	1.231356	1.1537 (1.081-1.2314)	0.041373859	0.008275
NSCLC	TCN2	Simple moc	21	0.128529	0.066917	6.91e-02	-0.00263	0.259686	1.137154	0.997375	1.296523	1.1372 (0.9974-1.2965)		
NSCLC	TCN2	Weighted n	21	0.131635	0.05024	1.64e-02	0.033164	0.230106	1.140692	1.03372	1.258734	1.1407 (1.0337-1.2587)		

NSCLC	LTB4R	MR Egger	7	-0.52086	0.282133	1.24e-01	-1.07384	0.032118	0.594008	0.341692	1.03264	0.594 (0.3417-1.0326)		
NSCLC	LTB4R	Weighted n	7	-0.36258	0.114067	1.48e-03	-0.58615	-0.13901	0.695877	0.556463	0.870219	0.6959 (0.5565-0.8702)		
NSCLC	LTB4R	Inverse vari	7	-0.40511	0.092179	1.11e-05	-0.58578	-0.22444	0.666901	0.556669	0.798961	0.6669 (0.5567-0.799)	0.02726776	0.007154
NSCLC	LTB4R	Simple moc	7	-0.31377	0.155137	8.96e-02	-0.61784	-0.0097	0.730685	0.539107	0.990343	0.7307 (0.5391-0.9903)		
NSCLC	LTB4R	Weighted n	7	-0.36116	0.117007	2.15e-02	-0.5905	-0.13183	0.696864	0.554051	0.876489	0.6969 (0.5541-0.8765)		

Table S2. Pleiotropy and heterogeneity tests of MR.

Outcome	Exposure	nsnp	egger_intercept	se_egger_intercept	pval_egger_intercept	Q_heterogeneity	Q_pval_heterogeneity
SCLC	PSMA4	7	0.034066153	0.109125831	0.767507735	7.712962317	0.259895619
SCLC	LTBP4	15	0.007376877	0.036827304	0.84433778	4.22713681	0.993937454
SCLC	MPI	11	-0.089824977	0.190423491	0.648358869	18.68786899	0.044411074
SCLC	TCN2	21	-0.059642228	0.060091187	0.333412539	25.38985995	0.186920966
SCLC	LTB4R	7	-0.079316806	0.077626318	0.353753344	4.933795445	0.552332035
NSCLC	PSMA4	7	0.063817783	0.067202107	0.38590383	23.77583294	0.00057423
NSCLC	LTBP4	15	0.013569177	0.019828031	0.505775957	27.65459005	0.015806096
NSCLC	MPI	11	-0.021102305	0.050021605	0.683022505	4.568944487	0.918054078
NSCLC	TCN2	21	-0.000887346	0.020023422	0.965115443	11.36943121	0.936073688
NSCLC	LTB4R	7	0.012675104	0.029199316	0.682316462	4.356387646	0.628566545

Table S3. The results of colocalization analysis.

exposure	outcome	SNPs	PP.H0.abf	PP.H1.abf	PP.H2.abf	PP.H3.abf	PP.H4.abf	PP.H4.abf_conditional	PP.H4.abf_1	PP.H4.abf_2
LTB4R	NSCLC	2069	4.74E-63	0.713322972	5.58E-64	0.083786762	0.202890266	0.707731161	0.286677028	2.421507412
LTBP4	NSCLC	1973	4.80E-192	0.021143696	4.75E-191	0.208632164	0.77022414	0.786861296	0.978856304	3.691780429
MPI	NSCLC	1566	3.78E-272	0.183629366	7.77E-272	0.377168029	0.439202605	0.537994125	0.816370634	1.164474639
PSMA4	NSCLC	2366	4.86E-129	1.44E-29	3.32E-100	0.985671107	0.014328893	0.014328893	1	0.014537195
TCN2	NSCLC	2117	0	0.801766754	0	0.100582576	0.097650669	0.492604905	0.198233246	0.970850745
PSMA4	SCLC	2366	1.08E-102	0.003205764	5.28E-101	0.156124848	0.840669387	0.843373043	0.996794236	5.384596987
